# The phylogenetic information carried by a new set of morphological characters in planthoppers: the internal mouthpart structures and test in the Cixiidae model (Hemiptera: Fulgoromorpha) 

**DOI:** 10.1007/s00435-013-0195-2

**Published:** 2013-05-23

**Authors:** Jolanta Brożek, Thierry Bourgoin

**Affiliations:** 1 Department of Zoology, University of Silesia, Bankowa 9, 40-007 Katowice, Poland; 2 Département Systématique and Evolution, Museum National d’Historie Naturelle, UMR 7205 MNHN–CNRS (ISEB), CP-50, 45 rue Buffon, 75005 Paris, France

**Keywords:** Fulgoromorpha, Cixiidae, Mouthparts, Internal connecting systems, Maxillary locks, Food and salivary canals

## Abstract

Internal morphological structures of Cixiidae mouthparts are described and compared in various representatives of the Cixiidae and several other representatives of hemipterans. The morphological study shows that the mouthpart structures have not evolved uniformly and reveals the great disparity of these structures. Particularly, the connecting system of the mouthparts, localisation of salivary canal and shape of the mandibular and maxillar stylets provide together a new set of 17 new characters. A parsimonious analysis to evaluate the phylogenetic interest carried by these 17 selected characters shows that mouthpart structures have not evolved anarchically, but that they indeed carry some phylogenetic information that will be useful to be included in further morphological phylogenetic analysis.

## Introduction

The Hemiptera are characterised by a deep modification of their buccal apparatus into a rostrum consisting of the labium guiding two pairs of respective mandibular and maxillar stylets allowing their penetration into feedings tissues. For mechanical efficiency, these stylets are morphologically more or less strongly coapted through interlocking devices. This mouthpart connecting system, which has been variously investigated according to the major Hemiptera taxa (Pollard 1968, 1972; Forbes and Raine 1973; Forbes 1977; Cobben 1978), has attracted new recent comparative analysis showing that it consists in a two- or three-locked system between the right and the left maxilla, surrounded by the two mandibles sometimes interlocked with the maxillae and the whole bunch being guided by the labium groove (Brożek and Herczek 2001, 2004; Brożek et al. 2006; Brożek 2006, 2007). Between the maxillary stylets, a dorsal alimentary and a ventral salivary canal are generally present.

A preliminary study of few representatives in some Hemiptera: Fulgoromorpha families has shown that the connecting system consists in a three-locked connecting system between the maxillae but also that some diversity in the shape of mandibles and maxillae should be of possible phylogenetic interest (Brożek et al. 2006). However, no further attempts were made to investigate more carefully these structures within a single family, and to evaluate how much these conformations observed were diverse at lower taxonomic levels. More particularly in Cixiidae, these first investigations have shown that the mandibulae were moon-crescent-shaped, of regular form (e.g. larger in cross-section in their mid-part) and joining dorsally and ventrally in a more or less rounded acute ending. A differently shaped system was observed in representatives of Delphacidae, Derbidae, Issidae, Caliscelidae and Lophopidae, which exhibited mandibles more developed ventrally (in cross-section) and with a wide ventral junction area. Moreover, as for Cixiidae, Issidae and Lophopidae maxillae were observed fully surrounded by the mandibles, while they were left freely exposed on their dorsal margin in all the other previously cited taxa and the Achilidae representative (Brożek et al. 2006).

Objectives in this study were therefore (1) to enlarge the scope of the morphological study of the mouthpart connecting system to some other planthopper families in order to better evaluate the interest of this new set of morphological characters for future phylogenetic studies in planthoppers; (2) to select a set of new identified characters and their states that should be useful to document in the future when describing new potential key taxa in the Fulgoroidea or even higher; and (3) to investigate, particularly within one planthopper family, whether any polymorphism of the connecting system is expressed as it is known to occur in Heteroptera for instance (Cobben 1978).

Until now, the Cixiidae monophyly still remains controversial and non-supported (Ceotto and Bourgoin 2008; Ceotto et al. 2008) and new character data sets are necessary to better assess this taxa. This is why the Cixiidae model was chosen as a good candidate to test the phylogenetic signal carried by the character selected in the labium in relation with the published internal classifications of the Cixiidae (Emeljanov 2002; Ceotto and Bourgoin 2008; Ceotto et al. 2008).

## Materials and methods

The study of the internal structures of the mouthparts was performed on dry material from the collections of the Museum National d’Historie Naturelle in Paris (MNHN) and of the Department of Zoology, University of Silesia, Katowice, Poland. The specimens are mainly Cixiidae, but several representatives of other family taxa were also included; all are listed in Appendix together with the species previously studied in other papers.

The internal structures of mouthparts were analysed through cross-section of the subapical labial segment of adult specimens. For scanning microscopy, the basal part of the head with a part of the labium was glued vertically, coated with a 65–70 μm film of gold–palladium and then photographed with a Jeol JSM III scanning electron microscope.

**Fig. 1:**
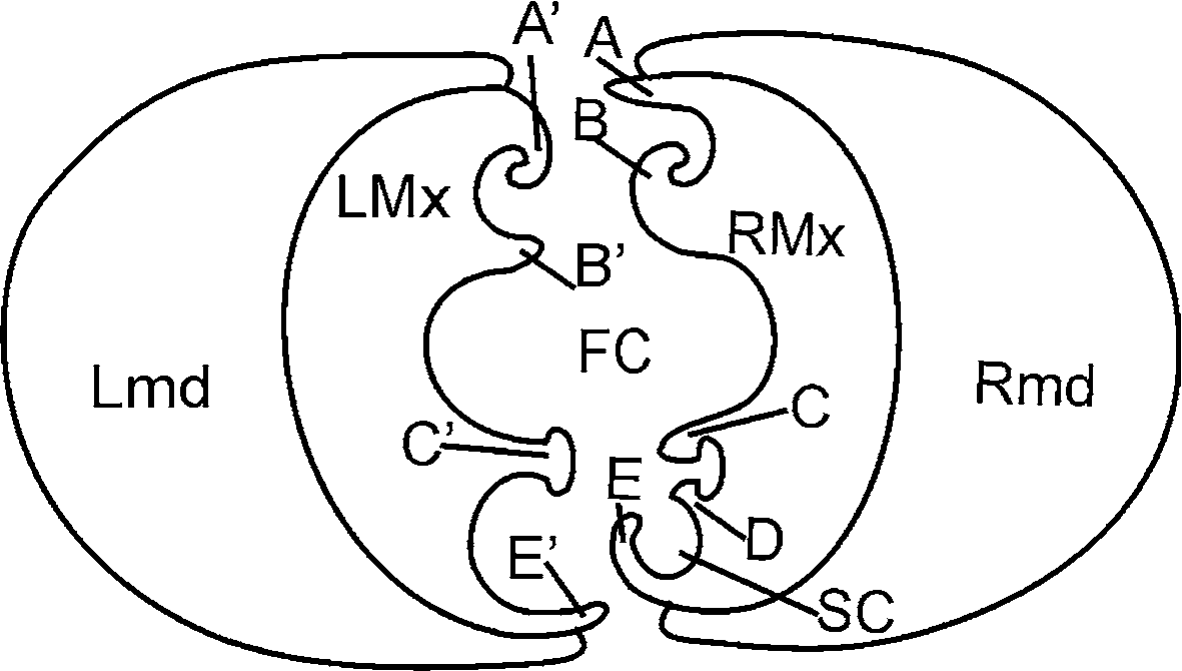
Model of cross-section through the subapical rostral segment of the Cixiidae: maxillae with three locks. *RMx* right maxilla, *LMx* left maxilla, *RMd* right mandible, *LMd* left mandible, *FC* food canal, *SC* salivary canal, *A* straight upper right process of the dorsal lock, *A′* hooked upper left process of the dorsal lock, *B* hooked lower right process of the dorsal lock, *B′* straight lower left process of the dorsal lock, *C* hooked upper right process of the middle lock, *C′* hooked upper left process of the middle lock, *D* hooked lower right process of the middle lock, *E* hooked lower right process of the ventral lock, *E′* hooked lower left process of the ventral lock

Terminology of the connecting system between maxillae and mandibles in the Cixiidae, at the level of the subapical segment, is presented in Fig. 1 and follows Brożek et al. (2006). Characters and states selected as of being of interest are noted [Kn (state number)] in the text. All of them are presented in Table 1 and have been illustrated with their different states in Fig. 11. All figures are presented in the apical view from the base to the apex of the rostrum with an indication of the dorsal, middle and ventral locks of the rostrum and with a 1 μm bar scale.

**Table 1:** Characters of interest

K1: Stylet bundle shape laterally compressed (higher than wider)/dorsoventrally compressed (wider than higher)/as wide as high/**0/1/2**
K2: Mandibular–maxillar interlocking device absent/present **0/1**
K3: Mandibular axis of greater width perpendicular to the dorsoventral axis/oriented lateroventral/oriented laterodorsal **0/1/2**
K4: Mandibles more than two (up to three) times longer than wide/less than two time as wide as long **0/1**
K5: Mandibular external margin regularly convex/concave laterodorsally/more complex and irregular **0/1/2**
K6: Mandibular external laterodorsal slip absent/present **0/1**
K7: Mandibular dorsal tip acute/tapered/flattened short/flattened wide **0/1/2/3**
K8: Mandibular ventral tip acute/tapered/flattened short/flattened wide **0/1/2/3**
K9: Mandibular dorsal tips not in contact/in contact **0/1**
K10: Mandibular ventral tips not in contact/in contact **0/1**
K11: Interlocked maxillae in cross-section laterally compressed (oval)/rounded/cordiform/dorsoventrally compressed (oval) **0/1/2/3**
K12: Maxillar inner margins parallel to mandibular inner margins in cross-section/rotated left **0/1**
K13: Ventral right E maxillar process short/medium/long **0/1/2**
K14: Maxillar dorsal margin regularly convex/concave/mixed (convexo-concave) **0/1/2**
K15: Maxillar connecting system: three locking system/two locking system **0/1**
K16: Mandibular stylets mirror images of each another/not mirror images of each another **0/1**
K17: Salivary canal: in the left maxilla/formed by both maxilla/in the right maxilla **0/1/2**

The matrix (Table 2) analysis was performed using PAUP*4.0 (Swofford 1998) and TNT (Goloboff et al. 2008). All characters have been used as non-ordered, of equal weight with ACCTRAN transformation option. Character state analysis was performed using Mesquite 2.75 (build 564) (Maddison and Maddison 2011).

**Table 2:** Matrix of characters state of internal structures of the mouthparts of the hemipteran groups

Number of characters	12345678911111111
	01234567
Sternorrhyncha: *Orthezia urticae*	00100000000000010
Coleorrhyncha: *Xenophyes cascus*	11210010100002001
Heteroptera: *Pentatoma rufipes*	21000020000002001
Heteroptera: *Nepa* *cinerea*	11100000003000001
Tettigarctidae: *Tettigarcta crinata*	10010000003100101
Cicadellidae: *Ulopa reticulata*	10010000003100101
Delphacidae: *Peregrinus maidis*	10100013002000001
Achilidae: *Achilla marginatifrons*	10101111010000001
Achilidae: *Ballomarius kawandanus*	10001011110000001
Derbidae: *Diostrombus gangumis*	10100011002000001
Dictyopharidae: *Dictyophara europaea*	20100013002000001
Fulgoridae: *Calyptoproctus* sp.	20100013102000001
Meenoplidae: *Nisia nervosa*	10100013002001001
Ricaniidae: *Pochazia antica*	10101013002000001
Flatidae: *Flata pallida*	20101113001000001
Flatidae: *Flatida* sp.	10100013002000001
Tropiduchidae: *Trienopa paradoxa*	10100013001000001
Caliscelidae: *Ommatidiotus dissimilis*	10100013001000001
Lophopidae: *Lophops africana*	20100113000000001
Tropiduchidae: *Numicia hulstaerti*	10100013002001001
Tettigometridae: *Tettigometra sulphurea*	10000011010000001
Tettigometridae: *Hilda* sp.	10000011010000001
C/Borystheninae: *Borysthenes lacteus*	10000000101000001
C/Bothriocerinae: *Bothriocera* sp.	10001032100000001
C/Brixidiini: *Brixidia boukokoensis*	10000011100000001
C/Brixidiini: *Brixidia variabilis*	10000011100000001
C/Brixiini: *Brixia rose*	10000011110000001
C/Cixiini: *Achaemenes lokobenis*	10000021101010001
C/Cixiini: *Cixius nervosus*	10000021100000001
C/Cixiini: *Cixius cunicularius*	10000021100000001
C/Cixiini: *Macrocixius giganteus*	10000021110000001
C/Cixiini: *Tachycixius pilosus*	10000021110000001
C/Oecleini: *Mundopa kotoshonis*	10000032110000001
C/Oecleini: *Myndus taffini*	10000032110000001
C/Pentastirini: *Oliarus kindli*	10000033100000001
C/Pentastirini: *Pentastiridius moestus*	10000031100000001
C/Pentastirini: *Hyalesthes obsoletus*	10000031100000001
C/Mnemosynini: *Mnemosyne camerunensis*	10000021110000001
C/Mnemosynini: *Mnemosyne lamabokensis*	10000021110000001
C/Pintaliini: *Cubana* sp.	10002111000020002
C/Pintaliini: *Pintalia* sp.	10001011000020002
C/Semonini: *Betacixius ocellatus*	10010012100010001

## Results

### Stylet bundle

Cross-sections through the stylet bundle in toto (interlocked maxillae surrounded by the two mandibulae) show that in all cixiid studied, the stylet bundle is distinctly dorsoventrally compressed [K1(1)]. In few cases (*Borysthenes* and *Achaemenes*), it even appears to be almost twice as wide as high.

### Mandibular–maxillar and maxillar–maxillar interlocking systems

In all the specimens examined, the mouthpart interlocking/connecting apparatus consists of a three-locked maxillar–maxillar system—dorsal, median and ventral—between the right (RMx) and the left (LMx) maxilla, surrounded by the two mandibles; the whole bunch surrounded by the labium (Fig. 1). The mandibles (RMd, LMd) are placed laterally with respect to the maxillae. Special device to interlock the mandibles with the maxillae (Fig. 11 [K2(1)], IMMD) was not observed [K2(0)], and the regularly convex external walls of the maxillae are able to slide along the concave internal and smooth surfaces of the mandibles. However, most often, the general shape of the interlocked maxillae prevents their free rotation within the case surrounded by the two mandibular stylets (see further in the “Discussion”).

### Mandibulae

**Fig. 2:**
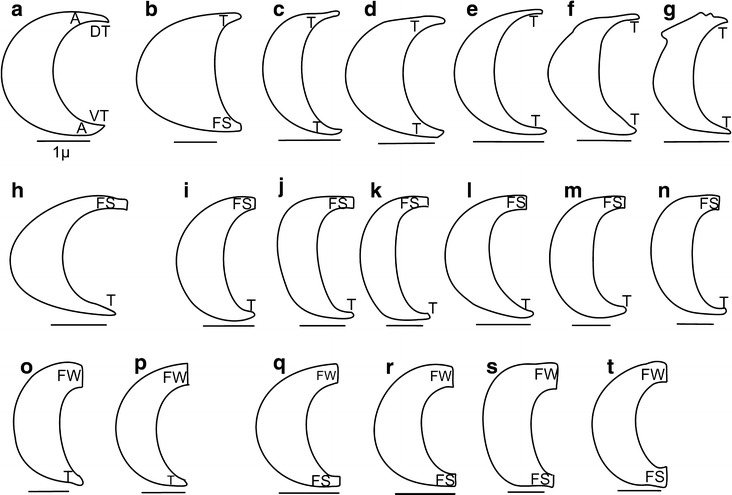
Types of the mandible shapes in the cross-section in the Cixiidae: **a** *Borysthenes lacteus*. **b** *Betacixius ocellatus*. **c** *Brixidia boukokoensis*. **d** *Brixidia variabilis*. **e** *Brixia rosae*. **f** *Pintalia* sp. **g** *Cubana* sp. **h** *Achaemenes lokobensis*. **i** *Cixius nervosus*. **j** *Cixius cunicularius*. **k** *Macrocixius giganteus*. **l** *Tachycixius pilosus*. **m** *Mnemosyne camerunensis*. **n** *Mnemosyne lamabokensis*. **o** *Pentastiridius moestus*. **p** *Hyalesthes obsoletus*. **q** *Mundopa kotoshonis*. **r** *Myndus taffini*. **s** *Bothriocera* sp. **t** *Oliarus kindli.* *DT* dorsal tip, *VT* ventral tip, *A* acute, *T* tapered, *FS* flattened short, *FW* flattened wide

In cross-section, mandibular stylets are more or less crescent-shaped and are a mirror image to each other [K16(0)]. They exhibit a high disparity of shapes (Fig. 2) that allow to recognise several specific mandibular characters for their description. Global shape: axis of their greater width appears to be perpendicular to the dorsoventral axis in all cixiids studied, tettigometrids and the achillid *Ballomarius* (Figs. 10a, 11 [K3(0)]) versus oriented lateroventral in all other Fulgoroidea studied (Figs. 10b, 11 [K3(1)]). Global development: in general, the mandibles are more than two to three times longer than wide [K4(0)]. In *Betacixius ocellatus,* they are distinctly wider, less than two time as wide as long [K4(1)]. Laterodorsal external margin shape: generally regularly convex [K5(0)] in most cixiids versus slightly concave laterodorsal [K5(1)] as in *Pintalia* sp., *Bothriocera* sp., achilids, the flatid *Flata* and the ricaniid *Pochazia* or even more complex as in *Cubana* sp., [K5(2)]; and with a latero-concave slip (sl) [K6(1)] as observed in *Cubana* sp., *Achilla marginatifrons, Flata pallida* or *Lophops africana*. The shape of the dorsal and ventral tips can be acute, tapered, flattened short or flattened wide [K7, K8].

**Fig. 3:**
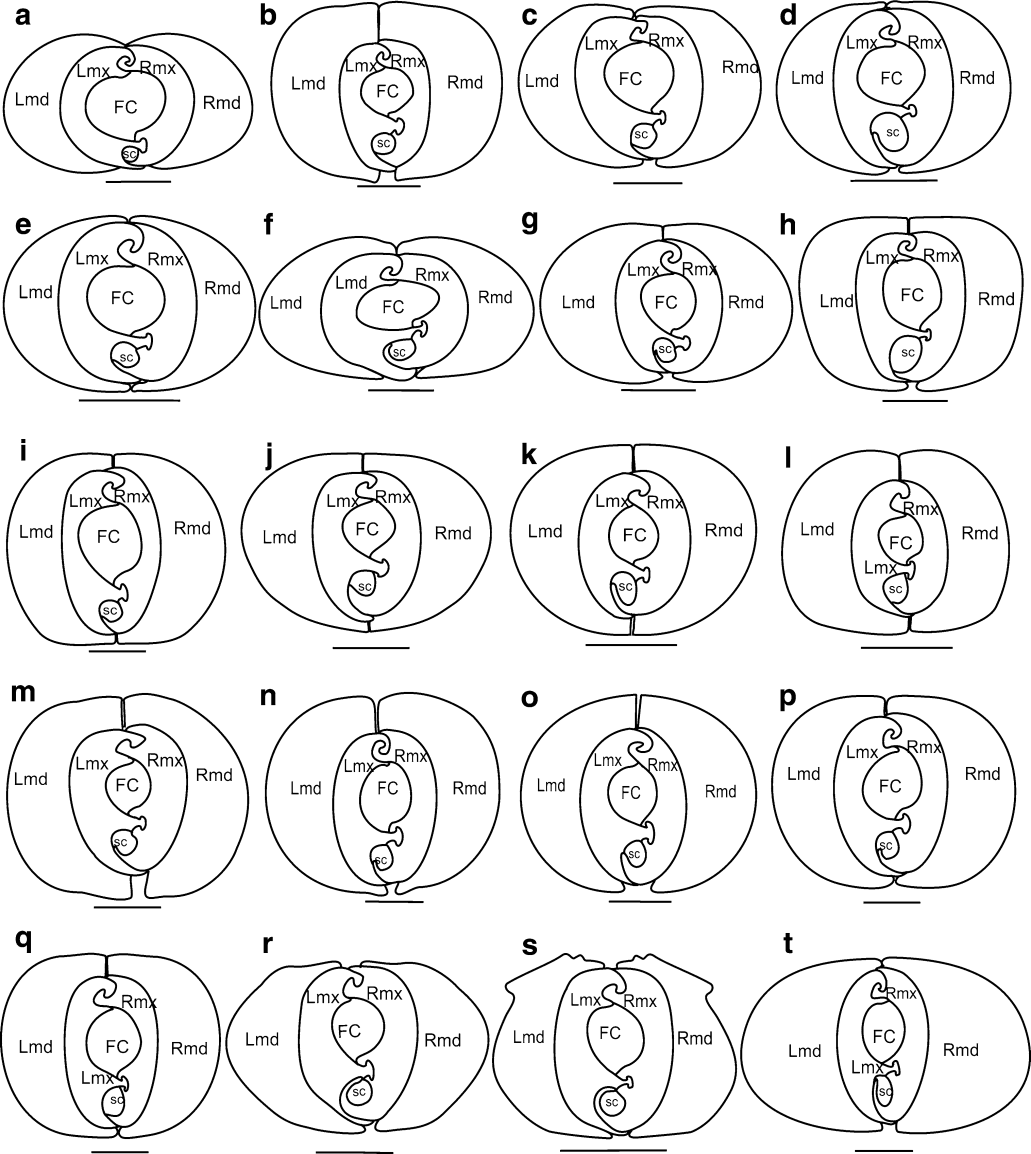
Shapes of the mandibles and maxillae in various cixiid representatives: cross-section through the subapical rostral segment. **a** *Borysthenes lacteus* (Borystheninae). **b** *Bothriocera* sp. (Bothiocerinae). **c** *Brixidia boukokoensis.* **d** *Brixidia variabilis* (Cixiinae: Brixidiini). **e** *Brixia rosae* (Cixiinae: Brixiini). **f** *Achaemenes lokobensis.* **g** *Cixius nervosus.* **h** *Cixius cunicularius.* **i** *Macrocixius giganteus.* **j** *Tachycixius pilosus* (Cixiinae: Cixiini). **k** *Mundopa kotoshonis.* **l** *Myndus taffini* (Cixiinae: Oecleini). **m** *Oliarus kindli.* **n** *Pentastiridius moestus.* **o** *Hyalesthes obsoletus* (Cixiinae: Pentastirini). **p** *Mnemosyne camerunensis.* **q** *Mnemosyne lamabokensis* (Cixiinae: Mnemosynini). **r** *Pintalia* sp. **s** *Cubana* sp. (Cixiinae: Pintaliini). **t** *Betacixius ocellatus* (Cixiinae: Semonini)

Almost all combinations between these last two characters have been observed: both dorsal and ventral tips acute (A) as in *Borysthenes lacteus* (Figs. 2a, 3a, 5a); dorsal tip tapered (T) and ventral tip flattened short (FS) as in *Betacixius ocellatus* (Figs. 2b, 3t, 6f); both dorsal and ventral tips tapered (T) as in *Brixidia boukokoensis* (Figs. 2c, 3c, 5c), *B. variablis* (Figs. 2d, 3d, 5d, e), *Brixia rosae* (Figs. 2e, 3e, 5f), *Pintalia* sp. (Figs. 2f, 3r, 6i) and *Cubana* sp. (Figs. 2g, 3s, 6k); dorsal tip flattened short (FS) and ventral tip tapered as in *Achaemenes lokobensis* (Figs. 2h, 3f, 5g), *Cixius nervosus* (Figs. 2i, 3g, 5h), *C. cunicularius* (Figs. 2j, 3h, 5i), *Macrocixius giganteus* (Figs. 2k, 3i, 5k), *Tachycixius pilosus* (Figs. 2l, 3j, 5l), *Mnemosyne camerunensis* (Figs. 2m, 3p, 6g) and *M. lamabokensis* (Figs. 2n, 3q, 6h); dorsal tip flattened wide (FW) and ventral tip tapered as in *Pentastiridius moestus* (Figs. 2o, 3n, 6d) and *Hyalesthes obsoletus* (Figs. 2p, 3o, 6e); dorsal tip flattened wide and ventral one flattened short as in *Mundopa kotoshonis* (Figs. 2q, 3k, 6a), *Myndus taffini* (Figs. 2r, 3l, 6b) and *Bothriocera* sp. (Figs. 2s, 3b, 5b); or both dorsal and ventral tips flattened wide as observed only in *Oliarus kindli* (Figs. 2t, 3m, 6c).

Finally, the two mandibles can be or not in contact both dorsally [K9] and ventrally [K10]. When dorsal tips are flattened, the two mandibular stylets are always dorsally in contact in Cixiidae (Fig. 3b, g–q). Similar junction was not observed in other planthoppers. When dorsal tips are acute or tapered, they might be in contact as in *Borysthenes*, *Brixidia*, *Bixia*, *Achaemenes* and *Betacixius* (Fig. 3a, c–f, t) or not as in *Pintalia* and *Cubana* (Fig. 3r, s,). Ventrally and whatever their shapes, ventral tips are generally not in contact with Cixiidae excepted in *Brixia*, *Macrocixius*, *Tachycixius*, *Mundopa*, *Myndus* and *Mnemosyne* (Fig. 3e, i–l, p, q).

### Maxillae

In cross-section, maxillae in Cixiidae are generally flattened laterally, together representing a more or less oval assemblage [K11(0)] higher than wide (Fig. 3b–d, g–t). The assemblage looks almost rounded [K11(1)] in the cixiid *Borysthenes* (Fig. 3a) and *Achaemenes* (Fig. 3f) as well as in some other planthoppers such as the tropiduchid Trienopa (Figs. 7g, 8i) or the flatid *Flata pallida* (Figs. 7i, 8j) or the caliscelid *Ommatidiotus* (Fig. 7l). In non-cixiid planthoppers observed, the two interlocked maxillae are also laterally compressed in the Tettigometridae (Figs. 7a, b, 8a, b), Achilidae (Figs. 7c, d, 8c, d) and Lophopidae (Figs. 7p, 9f), but in most cases, they form a cordiform assemblage more acute ventrally than dorsally [K11(2)] (Figs. 7e, f, h, j, k, m–o, 8f–h, k, l, 9a–e). In *Nisia* and *Numicia* (Figs. 7e, f, 8f, h), the dorsal margin is distinctly concave [K14(1)].

Between them, the maxillae delimit two canals: the ventral salivary canal (SC) more or less fully included in the right maxilla and the dorsal alimentary or food canal (FC), which is wider and formed by the junction of the two maxillar stylets. These are joined along their entire length through the connecting apparatus. In all cixiids and planthoppers studied, in cross-section, the junction line between the two maxillae runs parallel to the dorsoventral axis [K12(0)].

### Maxillar connecting apparatus

It is formed by a triple interlocking complex structure [K15(0)] of special internal arms bringing together variously shaped maxillar ridges (in this study referred to as processes) and grooves (Fig. 1):

1. Four processes form the dorsal lock. On the left maxilla: the upper hooked one (A′) and the lower straight one (B′), and on the right maxilla: the upper straight one (A) and the lower hooked one (B). Between A and B′, A′ and B interlock (Fig. 1).
2. The median lock is formed by three processes: the two hooked processes (C, D) of the right maxilla interlock with a T-shaped process (C′) of the left maxilla (Fig. 1).
3. The ventral lock is formed only by two processes: the hooked E process on the right maxilla interlocks with the slightly hooked E’ process on the left maxilla (Fig. 1).

**Fig. 4:**
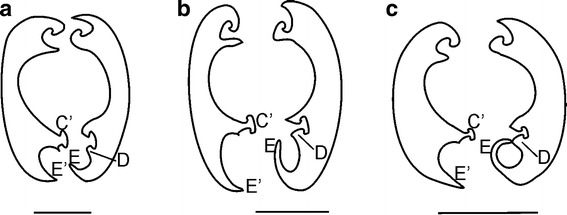
Length of the process E on the right maxilla of the Cixiidae. **a** Short, **b** middle, **c** long

**Fig. 5:**
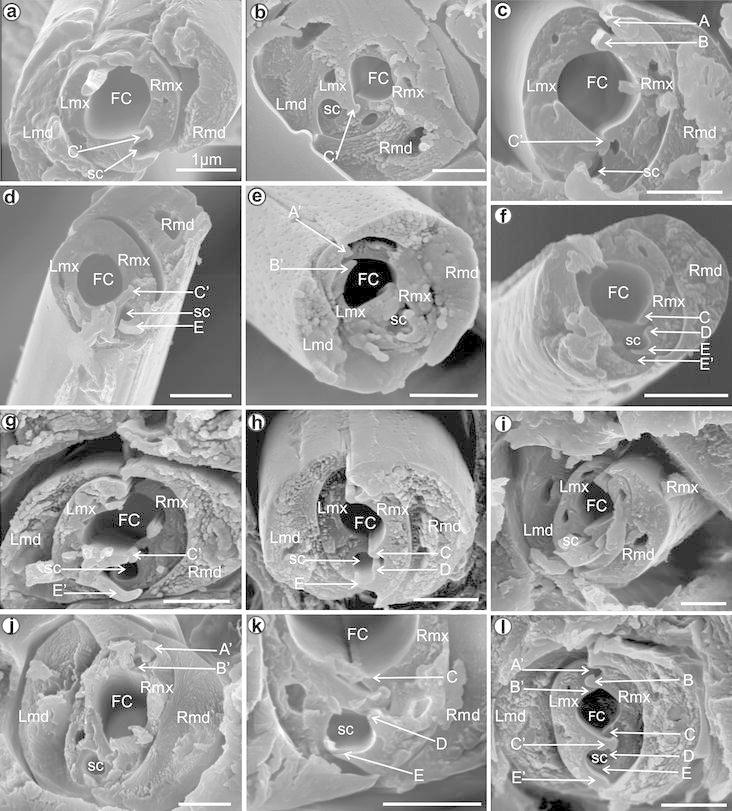
Detail of the cross-section through the subapical rostral segment of Cixiidae is presented based on scanning photos: **a** *Borysthenes lacteus* (Borystheninae). **b** *Bothriocera* sp. (Bothriocerinae). **c** *Brixidia boukokoensis*. **d** *B. variabilis* (Cixinae: Brixidiini). **e** *B. variabilis* (mandibles in contact). **f** *Brixia rosae* (Cixinae: Brixiini). **g** *Achaemenes lokobensis*. **h** *Cixius nervosus*. **i** *C. cunicularius*. **j** *Macrocixius giganteus*. **k** *M. giganteus* (middle lock is visible). **l** *Tachycixius pilosus*

**Fig. 6:**
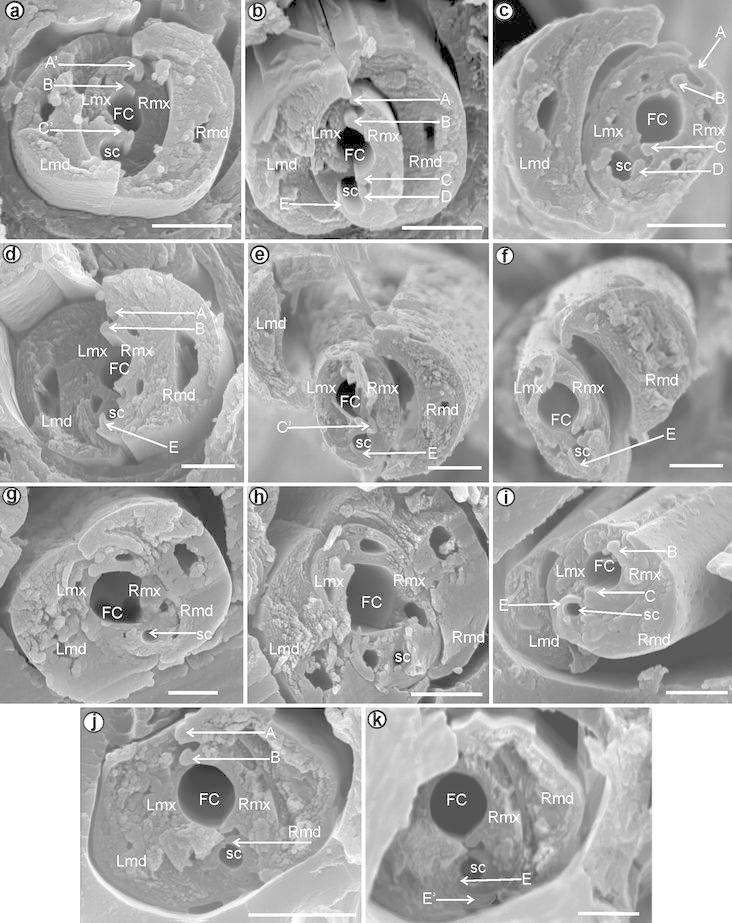
Detail of the cross-section through the subapical rostral segment of Cixiidae is presented based on scanning photos: **a** *Mundopa kotoshonis.* **b** *Myndus taffini* (Cixiinae: Oecleini). **c** *Oliarus kindli.* **d** *Pentastiridius moestus*. **e** *Hyalsethes obsoletus* (Cixiinae: Pentastirini). **f** *Betacixius ocellatus* (Cixiinae: Semonini). **g** *Mnemosyne camerunensis*. **h** *M. lamabokensis* (Cixiinae: Mnemosynini). **i** *Pintalia* sp. **j** *Cubana* sp. **k** *Cubana* sp. (processes E and E′ are visible) (Cixiinae: Pintaliini)
- A few intermediate situations (Fig. 4b) [K13(1)] were observed in *Achaemenes lokobensis* (Figs. 3f, 5g) and in *Betacixius ocellatus* (Figs. 3t, 6f) with a long E process only reaching the base of the C′ process.
- E process is hooked and long enough to reach the D process of the right maxilla [K13(2)] (Fig. 4c), and therefore, it encloses the whole salivary canal into the right maxilla [K17(2)] such as in the Pintaliini (*Pintalia* sp., *Cubana* sp.; Figs. 3s, r, 6i, j).

No variation was observed for the dorsal and median locks, while in the ventral one, the right E process appears to be more or less developed [K13] and therefore enclosing the salivary canal more or less completely:

- E process is considered as short [K13(0)] when its tip slightly overlaps the E′ process and does not reach up the base of the C′ process. In this conformation, the salivary canal is more or less equally closed by both the right and the left maxilla (Fig. 4a). This situation is observed in most cixiid species as in *Borysthenes lacteus* (Figs. 3a, 5a), *Bothriocera* sp. (Figs. 3b, 5b), *Brixidia boukokoensis, B. variabilis* (Figs. 3c, d, 5c–e) and *Brixia rosae* (Figs. 3e, 5f), and some Cixiini as in *Cixius nervosus, C. cunicularius, Macrocixius giganteus, Tachycixius pilosus* (Figs. 3g–j, 5h–l), the Pentastirini (*Oliarus kindli, Pentastiridius moestus, Hyalesthes obsoletus*, Figs. 3m–o, 4a), the Mnemosynini (*Mnemosyne camerunensis*, *M. lamabokoensis*, Figs. 3p, q, 6g, h) and the Oecleini (*Mundopa kotoshonis*, Figs. 3k, 6a; *Myndus taffini*, Figs. 3l, 6b).

## Discussion

The Hemiptera mouthpart connecting system has been scarcely investigated until now and only a few studies have been published on this subject (Cobben 1978; Pollard 1968, 1972; Forbes and Raine 1973; Forbes 1977). Recently, a comparative analysis of the systems has been undertaken (Brożek and Herczek 2004; Brożek et al. 2006; Brożek 2006, 2007). A surprising morphological diversity starts to emerge from these original studies, and a series of character appears to be of interest for further investigations. They will have to be included in morphological data set built for future phylogenetic analysis both from the Hemiptera level down to the family level, at least in planthoppers.

### Stylet bundle

**Fig. 7:**
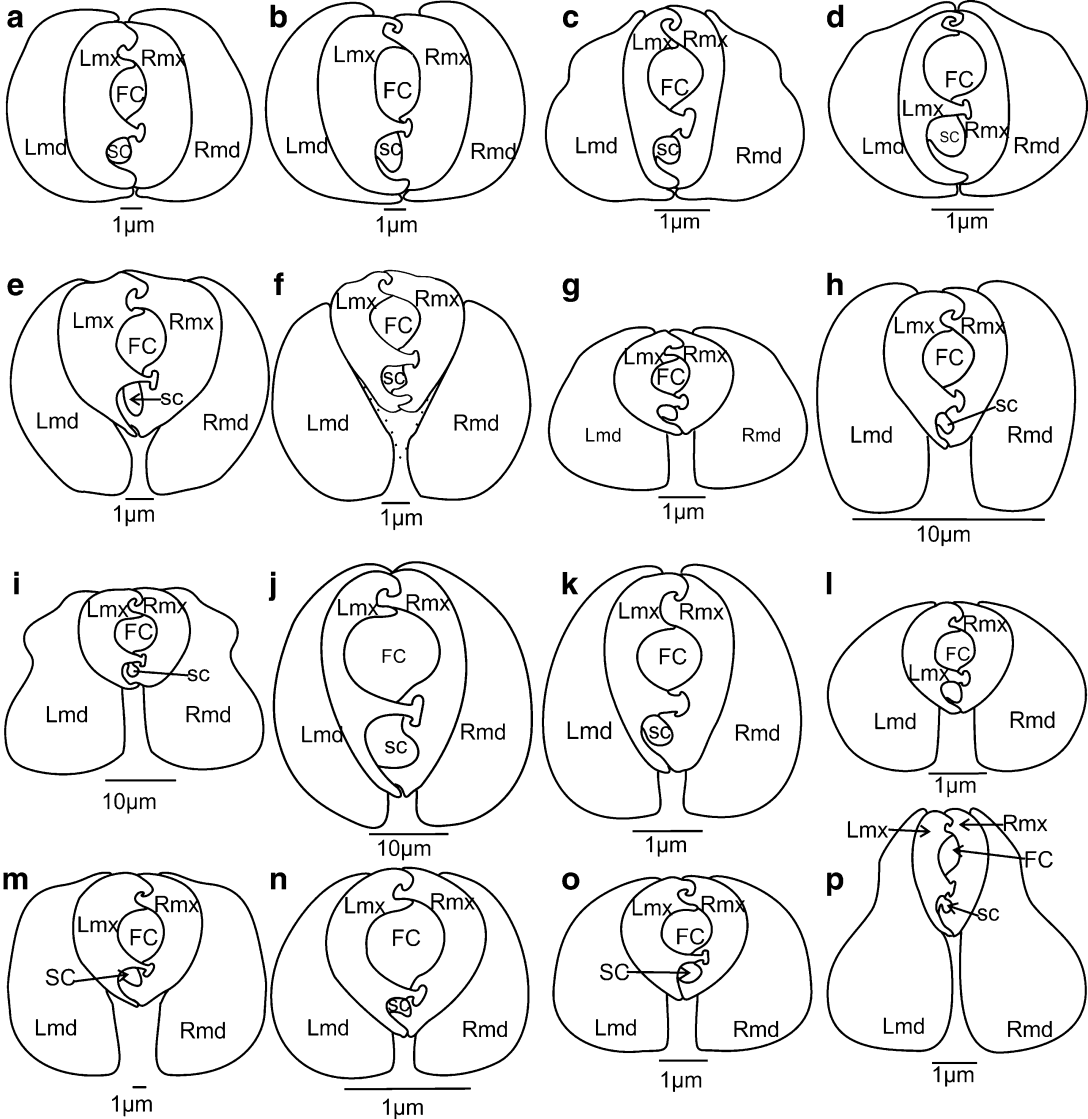
Shape of the maxillae and mandibles in cross-section of the representatives of the fulgoromorphan families: **a** *Tettigometra sulphurea*. **b** *Hilda* sp. **c** *Achilla marginatifrons*. **d** *Ballomarius kawandanus*. **e** *Nisia nervosa*. **f** *Numicia hulstaerti*. **g** *Trienopa paradoxa.* **h** *Flatida* sp. **i** *Flata pallida*. **j** *Calyptoproctus* sp. **k** *Dictyophara europaea*. **l** *Ommatidiotus dissimilis*. **m** *Pochazia antica*. **n** *Peregrinus maidis*. **o** *Diostrombus gangumis*. **p** *Lophops africana*. Abbreviations as on Fig. 1

In all Cixiidae studied, the stylet bundle is dorsoventrally compressed (wider than high) (Fig. 11. K 1(1)]). This is also the case in most planthoppers excepted in the representatives of dictyopharid, fulgorid and lophopid (Fig. 11. K 1(2)]) studied here. In flatids, the two states are observed: dorsoventrally compressed in *Flatida* sp. (Fig. 7h) and rounded in *Flata pallida* (Fig. 7i).

Some diversity is observed in other Hemiptera lineages: dorsoventrally compressed in Coleorrhyncha (Brożek 2007), in families of Cicadomorpha (Brożek in prep.) and most basal Heteroptera (Cobben 1978). It is of equal length (as wide as high) in Heteroptera Pentatomomorpha and Cimicomorpha (Brożek and Herczek 2004) or strongly laterally compressed (wider than high) in Sternorrhyncha (Fig. 11 [K1(0)]): Aphididae, Psyllidae and Aleyrodidae (Forbes 1969, 1972; Cobben 1978) and Coccinea (Brożek 2006).

As noted by Cobben (1978), no clear relation can be found between these different shapes of stylet bundle and feeding habits. The fact that all investigated cixiids and most other planthoppers are dorsoventrally compressed might indicate that this character state represents the plesiomorphic state.

### Mandibular–maxillar and maxillar–maxillar interlocking systems

For obvious functional reasons, maxillae and mandibulae have to maintain a smooth longitudinal slide between them, but efficiency of the system grows with the closer mechanical coordination of all the stylets due to additional interlocking devices. No such supplementary interlocking device [K2] was noticed between the stylets as it has been reported in many Heteroptera (Cobben 1978; Brożek and Herczek 2004). It would mean that in several cases, the mandibles might rotate around the maxillae at least on a short distance. However, this rotation remains blocked in most cases as soon as the concavity of the external maxilla margins is no more circular or if the maxillae are not symmetrical [K11].

This is particularly the case in most of the cixiids studied here, but also in delphacids, achilids, meenoplids, tettigometrids, tropiduchids, fulgorids, dictyopharids and lophopids representatives (Brożek et al. 2006). Only in the cixiid *Borysthenes*, and in *Flata pallida* (Flatidae), *Ommatidiotus* (Caliscelidae) and *Trienopa* (Tropiduchidae), circular interlocked maxillae have been observed (Brożek et al. 2006). Some limited rotation of the interlocked maxillae within the mandibular case might be possible for these taxa as also reported by Cobben (1978) for Gerromorpha even if in these taxa the lateral margin of the maxillae is irregular (op. cit. Fig. 143a, 147a, c) and the freedom of the connected maxillae is allowed by a slighter coordination of the mandibles and maxillae.

In other terms, planthoppers have developed a different morphological solution than in Heteroptera for the maxillar–mandibular interlocking system, without additional morphological device but just through a shape modification of their stylets. This planthopper system remains plesiomorphic and is much probably less efficient than the heteropteran one where these maxillar–mandibular locking have evolved.

### Mandibulae

As already mentioned by Cobben (1978), the mirror image of the two mandibules [K16 (0)] is a general condition for the Hemiptera excepted in the Sternorrhyncha [K16 (1)]. In this respect, and following Cobben hypothesis (1978: 236) that each mandibular stylet have evolved in opposite direction (or that one mandibule has evolved with some special adaptation), planthoppers share a probable plesiomorphic conformation with the other Auchenorrhyncha, Coleorrhyncha and Heteroptera. In planthoppers, they are usually more or less crescent-shaped while they exhibit a high disparity of shapes. In all Cixiidae studied, stylets are regularly convex on their external margin with their wider development passing through an axis perpendicular to the dorsoventral one (Figs. 10a, 11) [K3(0)].

**Fig. 8:**
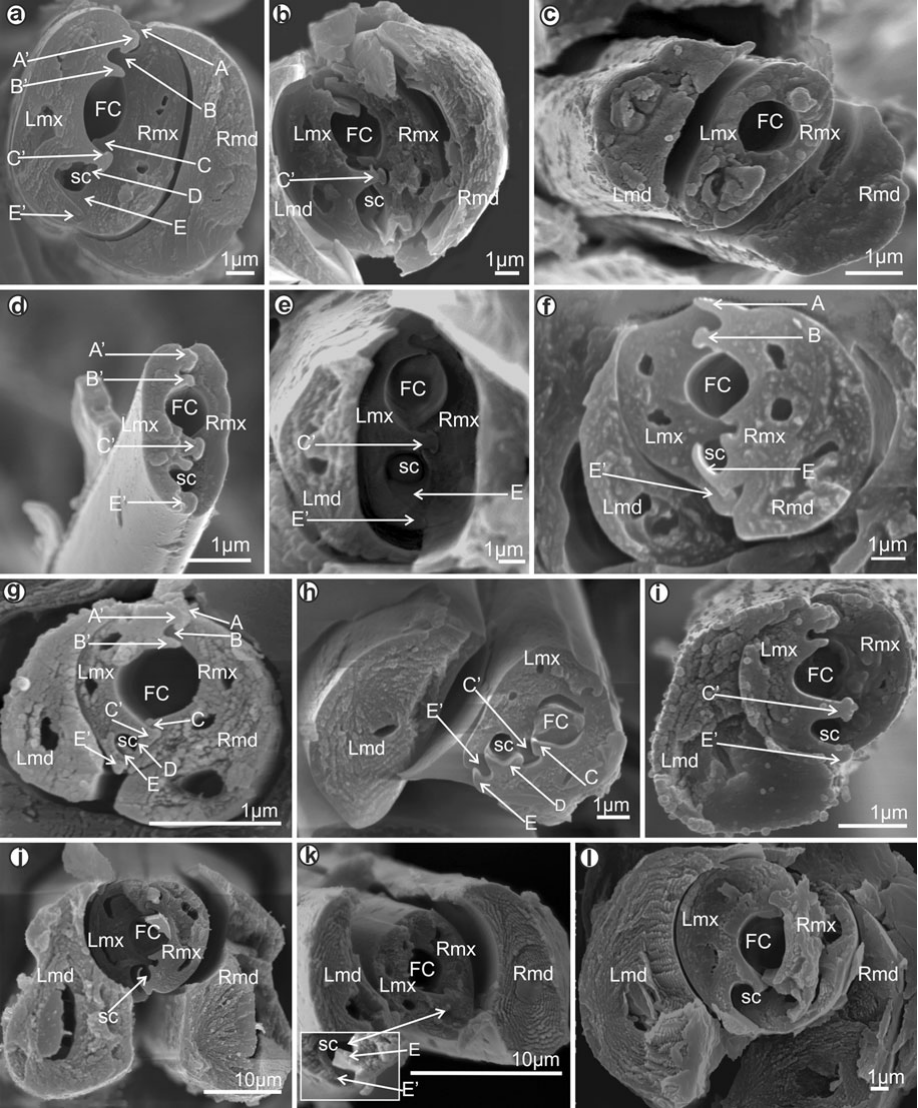
Cross-sections through the subapical rostral segment of the fulgoromorphan families: **a** *Tettigometra sulphurea* (Tettigometridae). **b** *Hilda* sp. (Tettigometridae). **c** *Achilla marginatifrons* (Achilidae). **d** *A. marginatifrons* (Achilidae). **e** *Ballomarius kawandanus* (Achilidae). **f** *Nisia nervosa* (Meenoplidae). **g** *Peregrinus maidis* (Delphacidae). **h** *Numicia hulstaerti* (Tropiduchidae). **i** *Trienopa paradoxa* (Tropiduchidae). **j** *Flata pallida* (Flatidae). **k** *Flatida* sp. (Flatidae). **l** *Pochazia antica* (Ricaniidae)

**Fig. 9:**
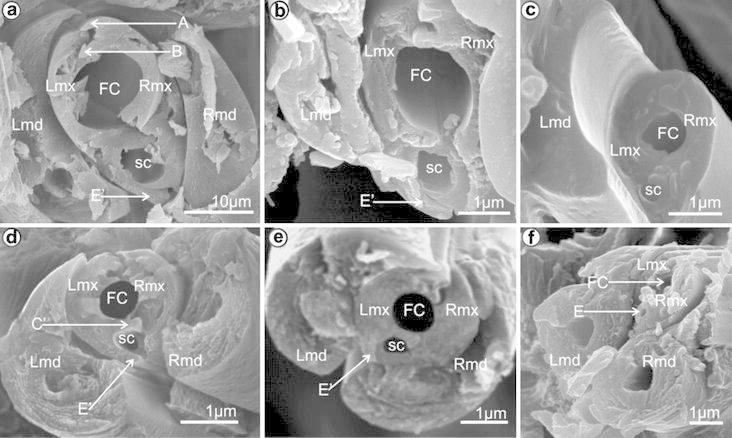
Cross-sections through the subapical rostral segment of the fulgoromorphan families: **a** *Calyptoproctus* sp. (Fulgoridae). **b** *Dictyophara europaea* (Dictyopharidae). **c** *D. europaea* (ventral lock is visible) (Dictyopharidae). **d** *Ommatidiotus dissimilis* (Caliscelidae). **e** *Diostrombus gangumis* (Derbidae). **f** *Lophops africana* (Lophopidae)

Excepted in the cixiid, the achilid *Ballomarius* and the tettigometrids, in all other planthoppers examined, the mandibulae use be more developed ventrally. The mandibulae is generally two to three times as high as wide [K4]; exceptionally in *Betacixius,* it is particularly wide, almost as it is high (Fig. 2b). In a few cixiid specimens (Fig. 2s) and in some other planthoppers, the dorsal marge [K5] is no more rounded, but concave as in achilids (Fig. 7c, d), flatid *Flata* sp. (Fig. 7i), ricaniid (Fig. 7m) and the lophopid (Fig. 7p) or irregular as in *Cubana* sp. (Fig. 2g, K5(2)). A distinct laterodorsal concave slip [K6] can be observed in *Cubana*, in the achilid *Achilla margninatifrons* (Fig. 7c), in the flatid *Flata pallida* (Figs. 7i, 8j.) and in the lophopid *Lophops* (Figs. 7p, 9f). This slip corresponds to two lateral ridges along the mandibles that probably help to guide the stylet bundle inside the labium but also prevent its rotation.

Ventrally or medially enlarged mandibles, as in the Fulgoromorpha (Brożek et al. 2006 and this study) and the Coleorrhyncha (Brożek 2007), and laterally flattened mandibles with undulated internal surface in Heteroptera (Cobben 1978; Brożek and Herczek 2004) represent probably an apomorphic condition with regard to the narrow and laterally flattened mandibles with smooth external and internal surface as one can observe in the Sternorrhyncha (Brożek 2006).

Dorsal and ventral tips of the mandibles vary in shape and in their mode of junction [K7, K8]. It is interesting to observe that in all cixiids, maxillae are almost fully surrounded by the mandibles [K9], while in most other planthoppers studied, the mandibles do not join dorsally, leaving free the dorsal margin of the interconnected maxillae (Fig. 7b, c, e–p). This condition is also general in Sternorrhyncha. As dorsally, the two mandibles might or not join ventrally [K10], even if the mandibles are strongly developed ventrally as in the flatids (Figs. 7i, 8j) for instance. Accordingly, maxillae almost fully surrounded by the mandibular case as in the Cixiidae might represent a synapomorphy for the taxa [K9]. The special case of the Pintalini as observed in *Pintalia* sp. and *Cubana* sp. represents probably another evolutionary step.

A tapered or flattened mandibular ventral tips seem to be a derived state in the Fulgoromorpha as not observed elsewhere in Hemiptera. In this respect, the particular condition observed in the one cixiid *Borysthenes* (acute ventral and dorsal tips) might be considered as autapomorphic reversals for each of these taxa.

### Maxillae

In cross-section, maxillae are generally flattened laterally, together representing a more or less oval assemblage [K11(0, 1)] longer than wide (Fig. 3b–e, g–t). As earlier mentioned, this transversal oval shape of the maxillae prevents the free rotation of the interlocked maxillar stylets inside the case formed by the two external mandibular stylets. In the other planthoppers observed, the two interlocked maxillae are also laterally compressed (Tettigometridae, Achilidae, Lophopidae) and in most cases form a cordiform assemblage [K11(2)]. In addition, the ventral development of the left maxillar stylet (E′ process) can also interlock ventrally between the two mandibles as in *Tachycixius* or *Achaemenes* where it appears to be more strongly developed. This double system prevents any rotation of the maxillae inside the mandibular case (Fig. 3f). This conformation participates to the interlocking apparatus and to the efficiency of the functionality of the connecting system.

It is interesting to note that in a few taxa as in the meenoplid *Nisia* (Figs. 7e, 8f) and the tropiduchid *Numicia* (Figs. 7f, 8h), the dorsal margin of the interlocked maxillae is widely exposed and concave [K14 (1)]. It is not known at present whether a corresponding labial structure exists.

**Fig. 10:**
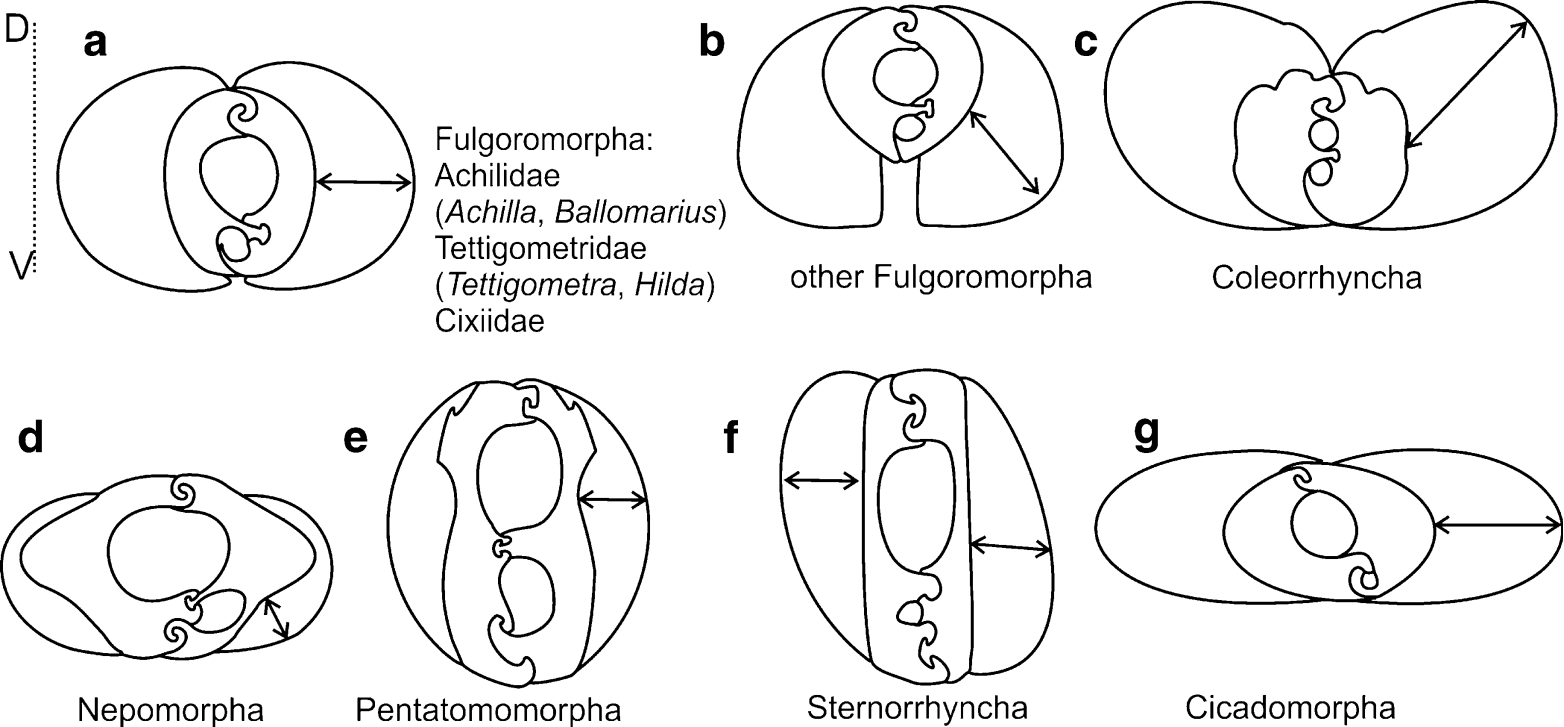
Axis of greater width in mandibles. **a** Fulgoromorpha (Achilidae, Tettigometridae, Cixiidae). **b** Other Fulgoromorpha. **c** Coleorrhyncha. **d** Heteroptera: Nepomorpha. **e** Heteroptera: Pentatomomorpha. **f** Sternorrhyncha. **g** Cicadomorpha. *D* Dorsal side, *V* ventral side

**Fig. 11:**
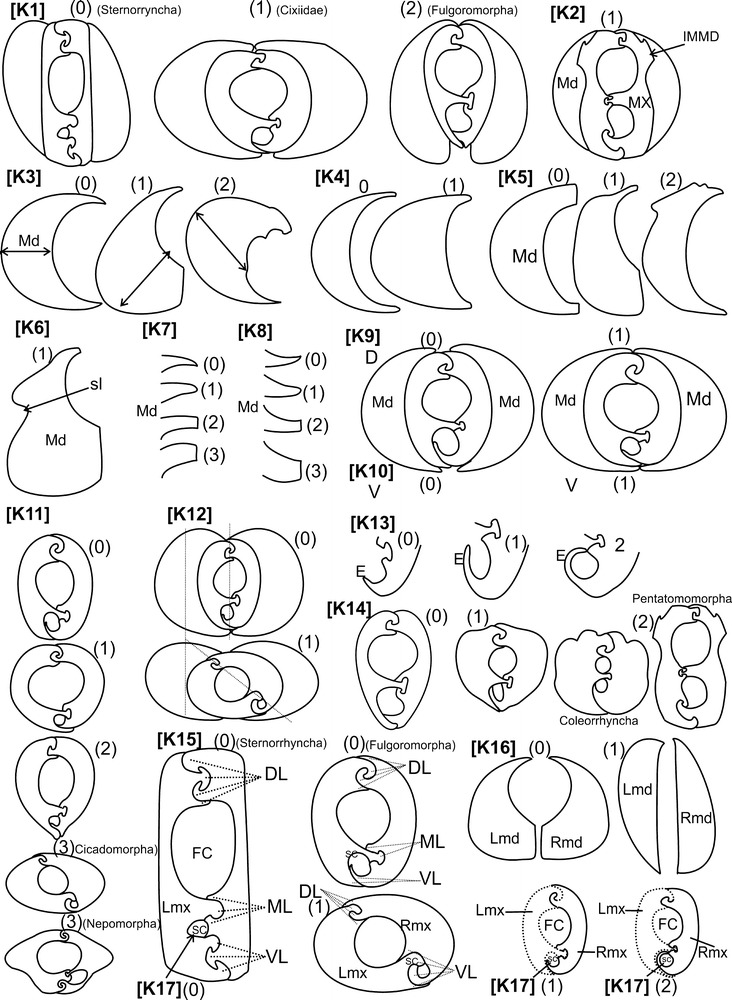
Characters states of the mandibles and maxillae, [K] as described in Table 1 with their states. *IMMD* interlocking mandibular–maxillar device

In all the planthopper taxa investigated (Brożek 2006, this study), the maxillar connecting system consists in a three-locking apparatus [K15(0)]. Such a condition appears to be plesiomorphic for the Hemiptera as exemplified in most Hemiptera: Sternorrhyncha (Pollard 1968; Forbes 1977; Cobben 1978; Brożek 2006), Fulgoromorpha (Brożek et al. 2006), and Coleorrhyncha and Heteroptera (Cobben 1978; Brożek and Herczek 2004; Brożek 2007) (Fig. 10a–e). Only in Cicadomorpha (Fig. 11, [K15(1)]), an apomorphic two locking system between the maxillae is observed (Brożek and Herczek 2001). This character is connected to the location of the salivary canal confined to the left maxillary stylet in Sternorrhyncha [K17(0)] as already documented by Cobben (1978).

In all Hemiptera, the two interconnected maxillae have in cross-section their inner margins parallel to the inner margins of mandibular stylets [K12(0)], but in the Cicadomorpha, the interconnected maxillae have rotated left and their junction is oblique compared to the dorsoventral axis presented by the two mandibles [K12(1)] as in Membracidae, Myerslopiidae and Aetalionidae in the proximal part of their stylets, and in Cercopoidea, Cicadoidea, Ledrinae (*Neotituria kongosana*), Iassinae (*Iassus lanio*), Idiocerinae (*Idiocerus stigmaticalis*) (Brożek in prep.) and in the Cicadellidae *Homalodisca* (Leopold et al. 2003, Fig. 21).

- Does the disparity of the mouth structures in observed Hemiptera and particularly planthoppers carry some phylogenetic signal useful for future evolutionary analysis?

In order to test the interest to develop further comparative morphological studies of the mouth structures for phylogenetic analysis, we have run a parsimonious analysis of the selected characters and states for all the taxa included in this study. Each of them represents a tribe in the Cixiidae or another planthopper family by including the previous data from Brożek et al. (2006). This analysis does not suppose to provide a phylogeny hypothesis of these taxa but rather to test whether the mouthpart complex allows some classification of these taxa into already recognised groups or whether the morphological message is too complex due to too much homoplasy, and therefore uninformative at this hierarchical level analysis. In other terms, this approach allows to observe and analyse the phylogenetic information of this set of characters restricted to the mouthparts as quoted in the matrix of Table 2, not being disturbed by the noise of the homoplasy carried by any other characters that will have been introduced into a parsimonious congruency analysis including other character sets. As one could expect it, the analysis did not produce any reliable result (too much taxa, too few characters) and resulted with strong polytomies. However, using Mesquite, we forced the tree topology to recover a classical Hemiptera phylogeny as in Bourgoin and Campbell (2002). On the base of a Sternorrhyncha Euhemiptera basal division and a polytomious Euhemiptera: Heteroptera, Coleorrhyncha, Cicadomorpha and Fulgoromorpha, the most parsimonious solution was therefore looking for within the Fulgormorpha and the Cixiidae.

**Fig. 12:**
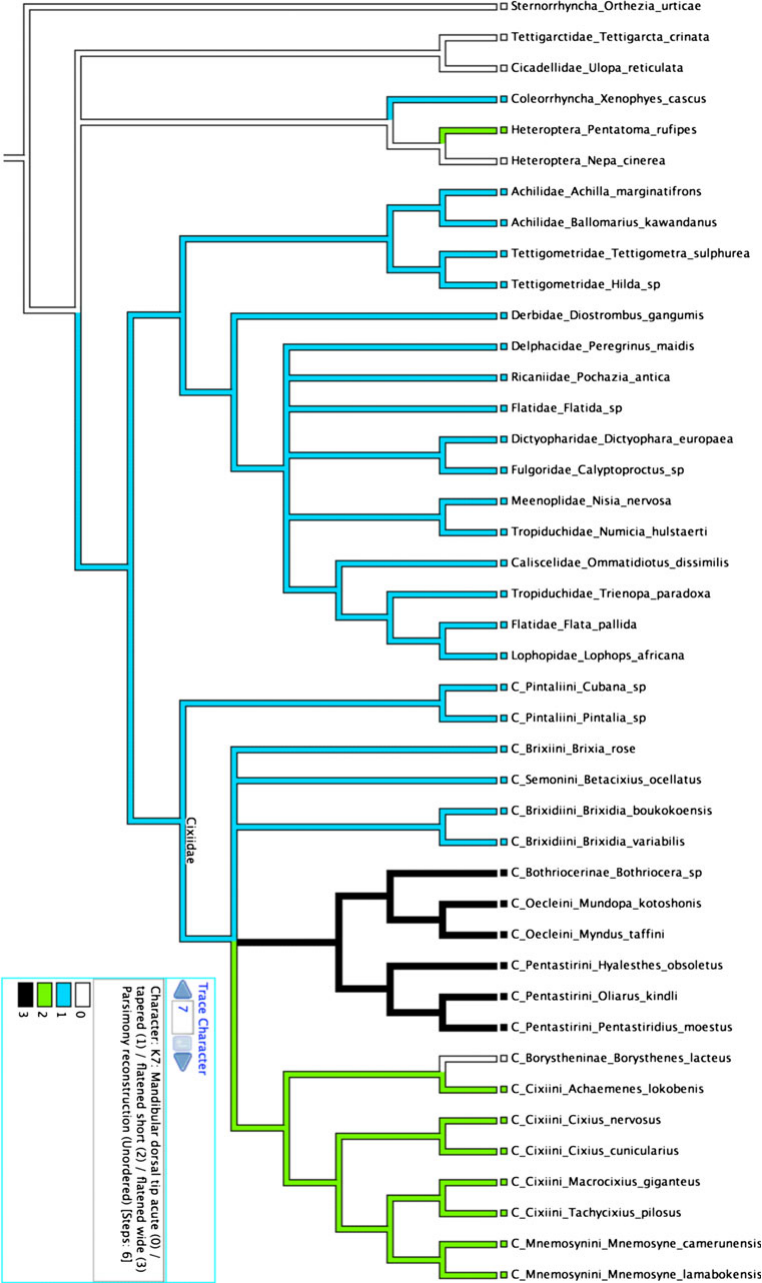
Parsimonious character state analysis of [mandibular dorsal tip] plotted on Hemiptera phylogeny according to Bourgoin and Campbell (2002)
- K8: all planthoppers except Cixiidae, Achilidae, Tettigometridae and Derbidae have a mandibular ventral tip tapered. In all other planthopper families, it is wide and flattened. A probable homoplasic conformation, flattened short is approached in Bothriocerinae and Oeclini (Fig. 13).

Obviously, some of these internal mouthpart characters appear to be of interest to be included in future morphological phylogeny studies of both Fulgomorpha and Cixiidae phylogenies. Particularly:

- K3: all planthoppers except Cixiidae, Tettigometridae and the achilid *Ballomarius* exhibit mandibular stylets more developed ventrally than dorsally.
- K7: all planthoppers including Cixiidae have a plesiomorphic mandibular dorsal tip tapered (Fig. 12). Within Cixiidae, Bothriocerinae, Oeclini and Pentastirini have a mandibular dorsal tip wide and flattened, while it remains short and flattened in all Cixiini.

**Fig. 13:**
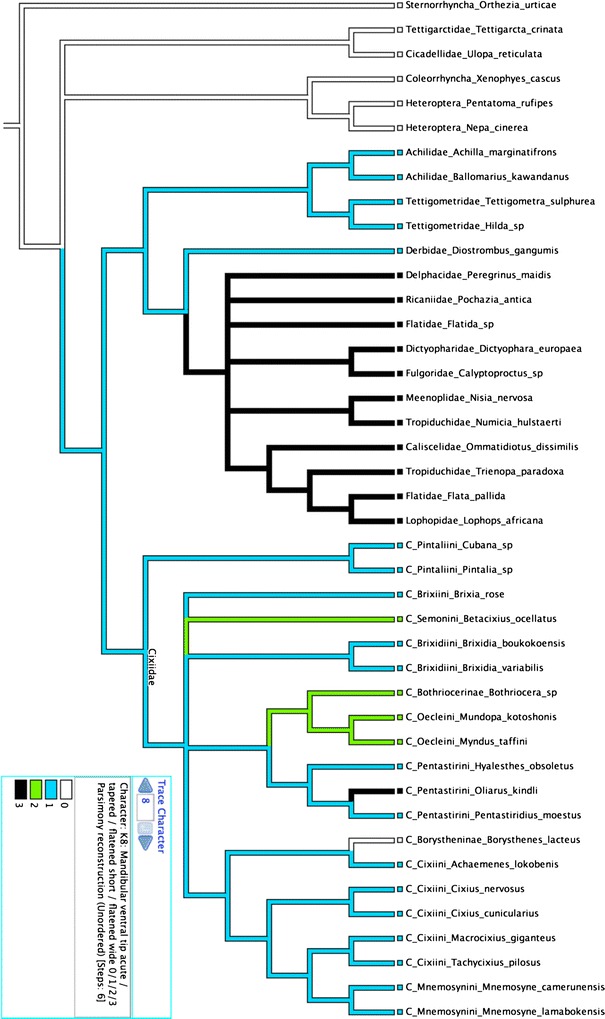
Parsimonious character state analysis of [mandibular ventral tip] plotted on Hemiptera phylogeny according to Bourgoin and Campbell (2002)
- K9: the mandibular case dorsally closed by joined mandibulae with the dorsal margin of maxillae not freely exposed represents a probable synapomorphy for all Cixiidae, excepted form the Pintalini where the dorsal margin of the mandibulae is not regularly convex (K5).

## Conclusions

The study has revealed an unexpected disparity of the mouthparts in the representatives of the different tribes of the Cixiidae, but also between them and some other planthopper families more generally. It shows the interest to investigate further these morphological diversities for future phylogenetic studies in planthoppers. Accordingly, a new set of identified characters and their states has been established (Table 1) to be documented in potential key taxa in the Fulgoroidea in the future. It is likely that it will have to be completed when more species will be examined.

The overall result does not contradict what it is generally admitted for the phylogeny of Fulgoromorpha (Bourgoin et al. 1997; Urban and Cryan 2007; Song and Liang 2013) and Cixiidae (Emeljanov 2002; Ceotto and Bourgoin 2008; Ceotto et al. 2008) even if none of these papers agrees together. The grouping together of most Cixiidae excepted in the Pintalini (which seems to exhibit several autapomorphies within the Cixiidae) versus the other planthoppers is congruent with a monophyletic Cixiidae taxa, as proposed by Emeljanov (2002) and Ceotto and Bourgoin (2008).

The study has shown that the evolution of the mouthpart structures does appears neither uniform nor anarchic, and that their study, extended to more taxa in other planthopper taxa, should deliver additional phylogenetic information that will be useful for future morphological phylogenetic studies of this group.

In the future, it will be interested to investigate further if these data, together with other mouthpart structures such as the recently studied labium sensilla in planthoppers (Brożek and Bourgoin 2013), could be linked to some possible diet structures or explain shift in patterns of trophic relationships as it has been observed in planthoppers (Attié et al. 2008).

## Appendix: List of studied Cixiidae specimens and others hemipteran

1. Borystheninae: *Borysthenes lacteus* Tsaur & Lee, 1987
2. Bothiocerinae: *Bothriocera* sp.
3. Cixiinae: Brixidiini: *Brixidia boukokoensis* Synave, 1980
4. Cixiinae: Brixidiini *Brixidia variabilis* Van Stalle, 1984
5. Cixiinae: Brixiini: *Brixia rosae* Synave, 1965
6. Cixiinae: Cixiini: *Achaemenes lokobensis* Synave, 1965
7. Cixiinae: Cixiini: *Cixius nervosus* (Linné, 1758)
8. Cixiinae: Cixiini *Cixius cunicularius* (Linné, 1767)
9. Cixiinae: Cixiini *Macrocixius giganteus* Matsumura, 1914
10. Cixiinae: Cixiini: *Tachycixius pilosus* (Olivier, 1791)
11. Cixiinae: Oecleini: *Mundopa kotoshonis* Matsumura, 1914
12. Cixiinae: Oecleini: *Myndus taffini* Bonfils, 1983
13. Cixiinae: Pentastirini: *Oliarus kindli* Bourgoin, Wilson & Couturier, 1998
14. Cixiinae: Pentastirini: *Pentastiridius moestus* (Stål, 1855)
15. Cixiinae: Pentastirini: *Hyalesthes obsoletus* Signoret, 1865
16. Cixiinae: Mnemosynini: *Mnemosyne camerunensis* Distant, 1907
17. Cixiinae: Mnemosynini: *Mnemosyne lamabokensis* Synave, 1979
18. Cixiinae: Pintaliini: *Cubana* sp.
19. Cixiinae: Pintaliini: *Pintalia* sp.
20. Cixiinae: Semonini: *Betacixius ocellatus* Matsumura, 1914
21. Tettigometridae: *Hilda* sp.
22. Tettigometridae: *Tettigometra sulphurea* Mulsant & Rey, 1855
23. Achilidae: *Achilla marginatifrons* Haglund, 1899
24. Achilidae: *Ballomarius kawandanus* Fennah, 1950
25. Meenoplidae: *Nisia nervosa* (Motschulsky, 1863)
26. Delphacidae: *Peregrinus maidis* (Ashmead) 1890)
27. Tropiduchidae: *Numicia hulstaerti* Synave 1962
28. Flatidae: *Flatida* sp.
29. Flatidae: *Flata pallida* (Olivier, 1791)
30. Ricaniidae: *Pochazia* *antica* (Gray, 1832)
31. Tropiduchidae (sensu Gnezdilov, 2007): *Trienopa paradoxa* (Gerstaecker, 1892)
32. Fulgoridae: *Calyptoproctus* sp.
33. Dictyopharidae: *Dictyophara europaea* (Linné, 1767)
34. Caliscelidae: *Ommatidiotus dissimilis* (Fallén, 1806)
35. Derbidae: *Diostrombus gangumis* Van Stalle, 1984
36. Lophopidae: *Lophops africana* (Schmidt, 1912)
37. Steronrrhyncha: *Orthezia urticae* (Linné, 1758)
38. Heteroptera: *Pentatoma ruphipes* (Linné, 1758)
39. Heteroptera: Nepa cinerea (Linné, 1758)
40. Coleorrhyncha: *Xenophyes cascus* Bergroth, 1924
41. Cicadomorpha: Tettigarctidae: *Tettigarcta crinita* Distant, 1883
42. Cicadomorpha: Cicadellidae: *Ulopa reticulata* Fabricius, 1794
